# Timing of Nasal Splint Removal After Septoplasty and Its Effects on Postoperative Functional Outcomes and Complications: A Systematic Review and Meta-Analysis

**DOI:** 10.7759/cureus.105960

**Published:** 2026-03-27

**Authors:** Lara S Alansari, Ahmed Almukhlifi, Ragad AlGarni, Ameneah Ayub, Yazeed Alosaimi, Meshal Almutairi, Shjoon Alnofaie, Walaa Al Khamis, Osama Alharbi, Norah Alkhashan, Judi Albishr, Rasil Alqahtani, Hadi Afandi Al-Hakami

**Affiliations:** 1 College of Medicine, King Saud Bin Abdulaziz University for Health Sciences, Jeddah, SAU; 2 College of Medicine, Taibah University, Medina, SAU; 3 Medicine and Surgery, Faculty of Medicine, King Abdulaziz University, Jeddah, SAU; 4 College of Medicine, Taif University, Taif, SAU; 5 College of Medicine, King Saud University, Riyadh, SAU; 6 College of Medicine, University of Bisha, Bisha, SAU; 7 College of Medicine, Ibn Sina National College for Medical Studies, Jeddah, SAU; 8 College of Medicine, Prince Sattam Bin Abdulaziz University, Al-Kharj, SAU; 9 College of Medicine, Jeddah University, Jeddah, SAU; 10 Otorhinolaryngology-Head and Neck Surgery, College of Medicine, King Saud Bin Abdulaziz University for Health Sciences, Jeddah, SAU

**Keywords:** bacterial colonization, nasal splint removal, postoperative complications, postoperative pain, septoplasty

## Abstract

The optimal timing for intranasal splint removal following septoplasty remains controversial. This systematic review and meta-analysis assessed how splint removal timing affects postoperative pain, nasal function, olfactory performance, and complications. In accordance with the Preferred Reporting Items for Systematic Reviews and Meta-Analyses (PRISMA) guidelines, we systematically searched PubMed, Google Scholar, and Web of Science (PROSPERO: CRD420251129055). Six studies from Turkey (2011-2024) involving 516 patients were included. Participants were stratified into early (days 1-3), intermediate (days 4-7), and late (>7 days) removal groups. Data were pooled using random-effects models. Results indicated that pain outcomes did not differ between early and intermediate removal, evidenced by a standardized mean difference (SMD) of 0.04 (p=0.80), but pain was significantly lower with late removal compared to intermediate timing (SMD 1.24, p<0.001). Nasal Obstruction Symptom Evaluation (NOSE) scores favored early (SMD -0.57, p=0.037) and intermediate (SMD -0.73, p=0.008) removal over late removal. Olfactory function, assessed using the Connecticut Chemosensory Clinical Research Center (CCCRC) odor test, was impaired with early removal compared to intermediate (SMD -0.56, p=0.039) and late removal (SMD -0.55, p=0.045). While complications showed no significant differences among groups, septal perforation risk was higher with early removal (OR 5.69, 95% CI 0.94-34.44, p=0.059), but this difference did not reach statistical significance.Notably, all included studies were conducted in Turkey, which may limit the generalizability of these findings to other populations and clinical settings*. *Ultimately, intermediate splint removal (days 4-7) may offer a favorable balance between pain control, functional recovery, and complication prevention following septoplasty.

## Introduction and background

Septoplasty is among the most frequently performed surgical procedures in otolaryngology, primarily indicated for correction of a deviated nasal septum, a major contributor to chronic nasal obstruction and impaired airflow [1,2]. Although the procedure is generally considered safe and effective, postoperative complications such as hemorrhage, septal hematoma, intranasal synechiae, and septal abscess may occur in approximately 3-7% of patients [3]. Dąbrowska-Bień et al. reported an overall complication rate of 3.42% in 5,639 patients, with adhesions, septal hematoma, and prolonged healing being most common [3]. To mitigate these risks, various intraoperative and postoperative measures have been adopted, with insertion of intranasal splints (INSs) being one of the most widely accepted techniques [4,5]. INSs play a crucial role in maintaining septal alignment and structural stability by preventing mucosal adhesion between opposing septal surfaces, reducing hematoma formation, and limiting dead space accumulation between subperichondrial layers [6].

Despite these benefits, splints may also contribute to postoperative discomfort, nasal obstruction, and pain during removal, as well as to potential complications such as mucosal dryness, crusting, septal perforation, and bacterial colonization [7,8]. While various studies have investigated different aspects of splint use, the optimal timing for splint removal and its relationship to postoperative outcomes remains unclear.

This systematic review and meta-analysis represents, to our knowledge, the first quantitative synthesis specifically examining how splint removal timing affects postoperative outcomes following septoplasty. The specific objectives are to: (1) compare pain outcomes across early (days 1-3), intermediate (days 4-7), and late (>7 days) removal timeframes; (2) evaluate functional outcomes, including subjective nasal obstruction and olfactory function; and (3) assess complication rates according to removal timing. By synthesizing the available evidence, this review aims to provide evidence-based guidance for clinical decision-making regarding the optimal timing of intranasal splint removal.

## Review

Methods

This systematic review and meta-analysis were conducted and reported in accordance with the Preferred Reporting Items for Systematic Reviews and Meta-Analyses (PRISMA) 2020 guidelines.

Protocol Registration

**Figure 1:**
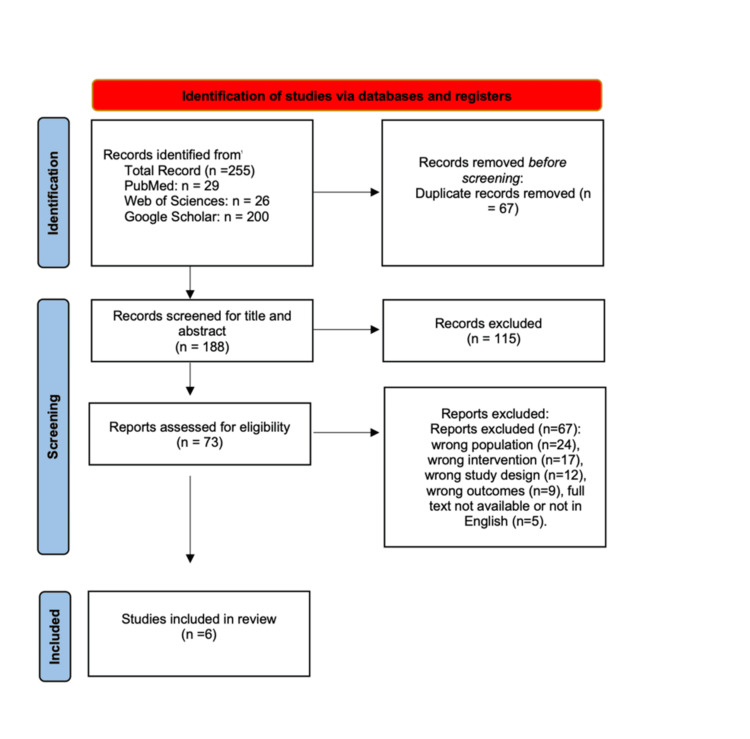
PRISMA flow diagram of study selection PRISMA: Preferred Reporting Items for Systematic Reviews and Meta-Analyses

The protocol was prospectively registered in the PROSPERO database (CRD420251129055) prior to study initiation. A PRISMA 2020 flow diagram (Figure 1) illustrates the study selection process. The protocol predefined the search strategy, eligibility criteria, data extraction process, and quality assessment methods.

Search Strategy Eligibility Criteria

A comprehensive literature search was performed in PubMed, Google Scholar, and Web of Science from database inception to August 8, 2025. The search combined both Medical Subject Headings (MeSH) terms and free-text keywords related to septoplasty, intranasal splints, timing of removal, and postoperative complications. The search strategy was adapted for each database. It included keywords such as ("septoplasty" OR "nasal surgery") AND ("intranasal splint" OR "nasal pack") AND ("removal time" OR "early removal" OR "delayed removal") AND ("complication" OR "pain" OR "bleeding" OR "adhesion" OR "bacterial colonization").

Eligibility criteria were defined using the PICOS framework: Population: patients undergoing septoplasty with postoperative intranasal splints; Intervention: early splint removal (postoperative days 1-3); Comparison: intermediate (days 4-7) and late removal (days >7); Outcomes: postoperative pain, nasal obstruction, olfactory function, bleeding, infection, adhesion formation, septal hematoma, bacterial colonization/biofilm formation, and patient satisfaction. The study included randomized controlled trials (RCTs) and comparative observational studies published in English.

Exclusion criteria included non-comparative designs, case reports, reviews, editorials, animal studies, and inaccessible full texts. Also, all references were managed using Mendeley Desktop version 1.19.8 (Elsevier, Amsterdam, the Netherlands, 2019) for storage, organization, and removal of duplicates.

Data Extraction and Standardization

All retrieved records were imported into a reference manager, and duplicates were removed. Titles and abstracts were screened independently by two reviewers against the predefined criteria, followed by full-text assessments; disagreements were resolved by a third reviewer.

Study Selection and Data Preparation

Data were extracted independently by two reviewers using a standardized, piloted data‑extraction form. For each included study, the following information was collected: study design, setting, sample size, patient demographics, type of surgery, splint type, timing of splint removal, follow‑up duration, and all reported outcomes (pain scores, Nasal Obstruction Symptom Evaluation (NOSE) score, olfactory function, Peak Nasal Inspiratory Flow (PNIF), saccharin transit time, and postoperative complications). When outcomes were reported at multiple time points, the earliest postoperative assessment within the first week was preferentially extracted for pain and subjective obstruction, while the longest available follow‑up was extracted for complications. Continuous outcomes reported in different scales were converted to standardized mean differences (SMDs), and where necessary, standard errors or confidence intervals were transformed into standard deviations using established formulas. When data were incomplete or unclear, corresponding authors were contacted for clarification. All extracted data were cross‑checked, and discrepancies were resolved by consensus.

Effect Size Calculation

Continuous outcomes were pooled using weighted means and standard deviations. SMDs with Hedges' g were calculated for continuous variables, with positive values indicating higher scores in later removal groups, computed as the mean of the first group minus the mean of the second group in each comparison, divided by the pooled standard deviation; thus, positive values indicate higher scores in the first (earlier removal) group and negative values indicate higher scores in the second (later removal) group. Binary outcomes were expressed as odds ratios (ORs), with a continuity correction of 0.5 applied for zero-event cells.

Subgroup Analyses and Meta-Regression Analysis

Subgroup analyses were performed according to splint removal timing (early, intermediate, late) and study design (RCT vs. observational). Meta-regression was planned to explore potential sources of heterogeneity, including patient age, surgical technique, and follow-up duration. Mixed groups containing multiple removal timepoints were pooled as composite interventions.

Statistical Analysis

Random-effects meta-analysis was performed using the DerSimonian-Laird method for binary outcomes and restricted maximum likelihood (REML) for continuous outcomes. Heterogeneity was assessed using the I^2 ^≤ statistic, categorized as low (0-25%), moderate (26-50%), substantial (51-75%), and considerable (>75%). Studies reporting a single outcome were included, with effect sizes and 95% confidence intervals reported, but not pooled. Publication bias was not assessed due to <10 studies per outcome. Analyses were performed using R (version 4.4.1; R Foundation for Statistical Computing, Vienna, Austria) with the meta and metafor packages with the "meta" package (version 8.2.1) and "metafor" package (version 4.6.0). Data manipulation was performed using "tidyverse" (version 2.0.0), and visualizations were created using "ggplot2" (version 3.5.1). Significance was set at p<0.05.with significance set at p<0.05.

Risk of Bias Assessment

The risk of bias for RCTs was assessed using the Cochrane Risk of Bias 2.0 tool (RoB-2), while observational studies were evaluated using the Risk Of Bias In Non-randomized Studies - of Interventions (ROBINS-I) tool. Two reviewers independently performed the assessments, with disagreements resolved by consensus or a third reviewer.

Results

Study Selection and Characteristics

The database search identified 255 records (PubMed n=29, Google Scholar n=200, Web of Science n=26). After removal of 67 duplicates, 188 unique titles and abstracts were screened, leading to the exclusion of 115 records. Of the 73 full-text articles assessed for eligibility, 67 were excluded for the following reasons: non-comparative study design, absence of relevant postoperative outcomes, inappropriate splint timing comparisons, duplicate or overlapping study populations, or insufficient data for extraction. The remaining six studies met all inclusion criteria and were included in the qualitative and quantitative synthesis (Figure 1).

All six included studies were conducted in Turkey between 2011 and 2024 and enrolled adult patients undergoing septoplasty for nasal septal deviation. Three were RCTs, and three were prospective or retrospective cohort studies. The pooled sample comprised 516 participants with a mean age of 32.3 years and a male predominance (62.4%). The studies varied in surgical technique, splint type, postoperative care protocols, and use of antibiotics. Details of study design, sample size, surgical technique, splint type, and timing of removal are summarized in Table 1 [7,9-13].

**Table 1:** Characteristics of the included studies

Study	Year	Country	Design	Population	Sample Size	Average Age
Bayram [12]	2022	Turkey	Prospective randomized clinical study	Patients who had septoplasty	84	32.2
Dag [9]	2014	Turkey	Prospective, non-randomized controlled study	Patients who had septoplasty	25	36.3
Aksoy [7]	2011	Turkey	Retrospective chart review	Patients who had septoplasty	96	32.4
Ozdogan [11]	2016	Turkey	Prospective clinical study	Patients who had septoplasty	109	32.3
Arslan [13]	2024	Turkey	Randomized controlled trial	Patients who had septoplasty	100	33.85
Karatas [10]	2016	Turkey	Prospective randomized clinical trial	Patients who had septoplasty	95	29.6 ± 7.8

Risk of Bias

**Figure 2:**
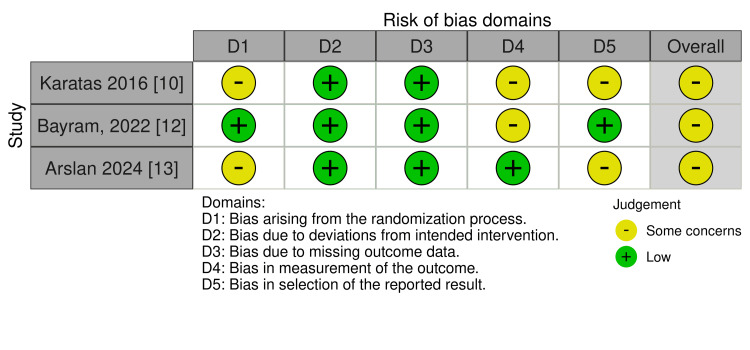
Risk of Bias 2 (RoB 2) traffic-light plot for the three randomized controlled trials (RCTs) This figure is original and was created by the authors from their risk‑of‑bias assessments; no previously published material has been reproduced. Studies included [10,12,13]

Risk of bias in randomized trials was evaluated with RoB‑2, and non‑randomized studies were assessed with ROBINS‑I (Figure 2 and Table 2). None of the RCTs were judged to be at an overall low risk of bias. All three trials showed some concerns, most commonly related to bias arising from the randomization process (D1) and, in one study, to outcome measurement (D4).

**Table 2:** Summary of risk-of-bias judgments for included studies RCT: randomized controlled trial; ROBINS-I: Risk Of Bias In Non-randomized Studies - of Interventions; RoB-2: Risk of Bias-2

Study	Design	Tool	Overall Risk of Bias	Main Concerns
Bayram 2022 [12]	RCT	RoB‑2	Some concerns	Randomization process; outcome measurement
Arslan 2024 [13]	RCT	RoB‑2	Some concerns	Randomization process
Karataş 2016 [10]	RCT	RoB‑2	Some concerns	Randomization process; outcome measurement
Dag 2014 [9]	Prospective cohort (non‑comparative)	ROBINS‑I	Moderate	Confounding; participant selection
Aksoy 2011 [7]	Retrospective cohort	ROBINS‑I	Serious	Confounding; selection; deviations; co‑treatments
Ozdogan 2016 [11]	Prospective cohort	ROBINS‑I	Moderate	Confounding; allocation method

**Figure 3:**
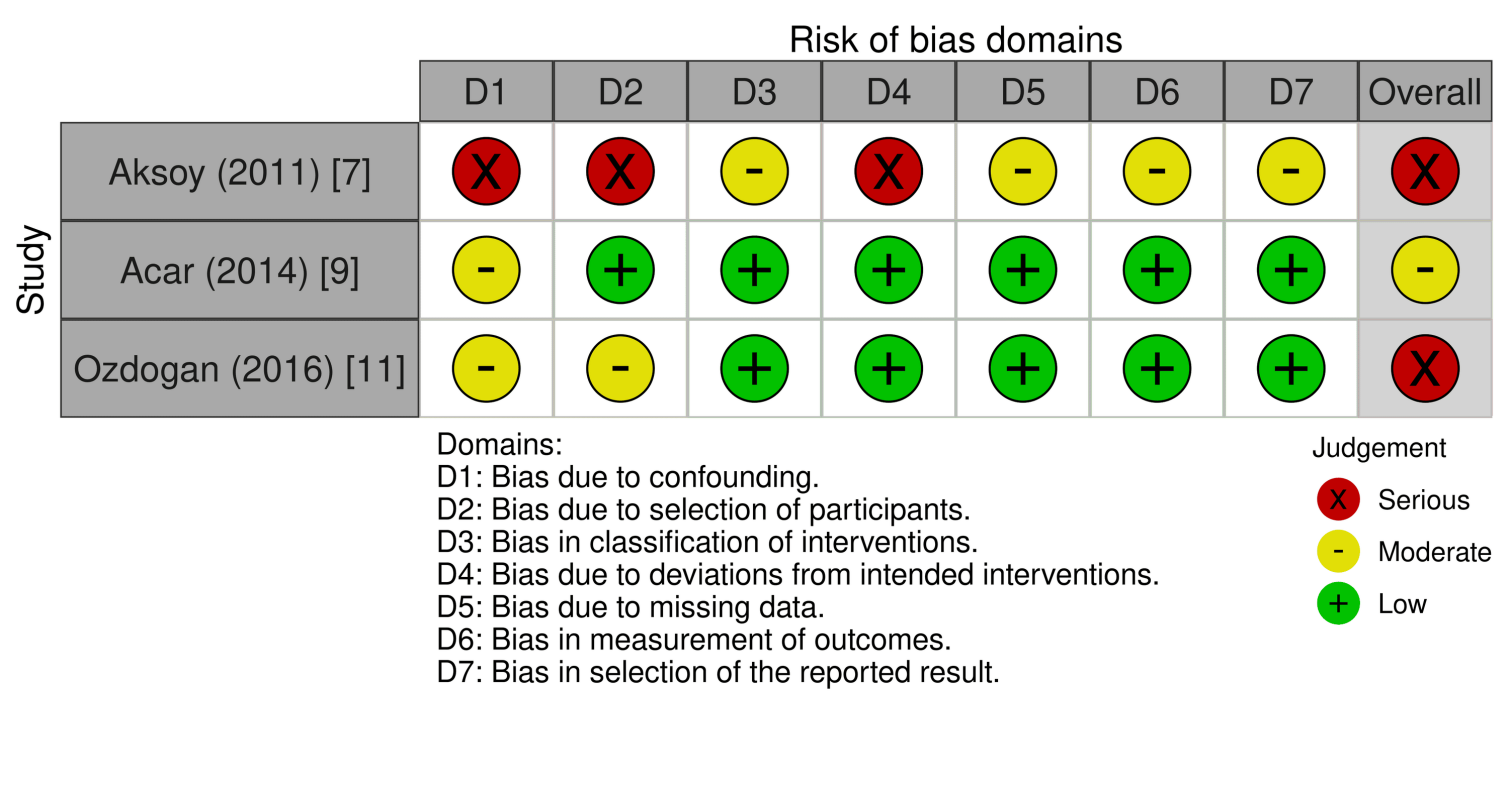
ROBINS-I traffic-light plot for the three non-randomized studies This figure is original and was created by the authors from their risk‑of‑bias assessments; no previously published material has been reproduced. Studies included [7,9,11] ROBINS-I: Risk Of Bias In Non-randomized Studies - of Interventions

Among the three non‑randomized studies, two (Dag 2014 and Ozdogan 2016) were rated at moderate overall risk of bias, reflecting potential confounding and limitations in participant selection, whereas one retrospective cohort study (Aksoy 2011) was judged to be at serious risk of bias due to non‑random allocation, substantial exclusions, and limited control for confounding and co‑interventions [7,9,5]. The detailed domain‑level judgments for the three non‑randomized studies are illustrated in Figure 3.

Continuous Outcomes

**Figure 4:**
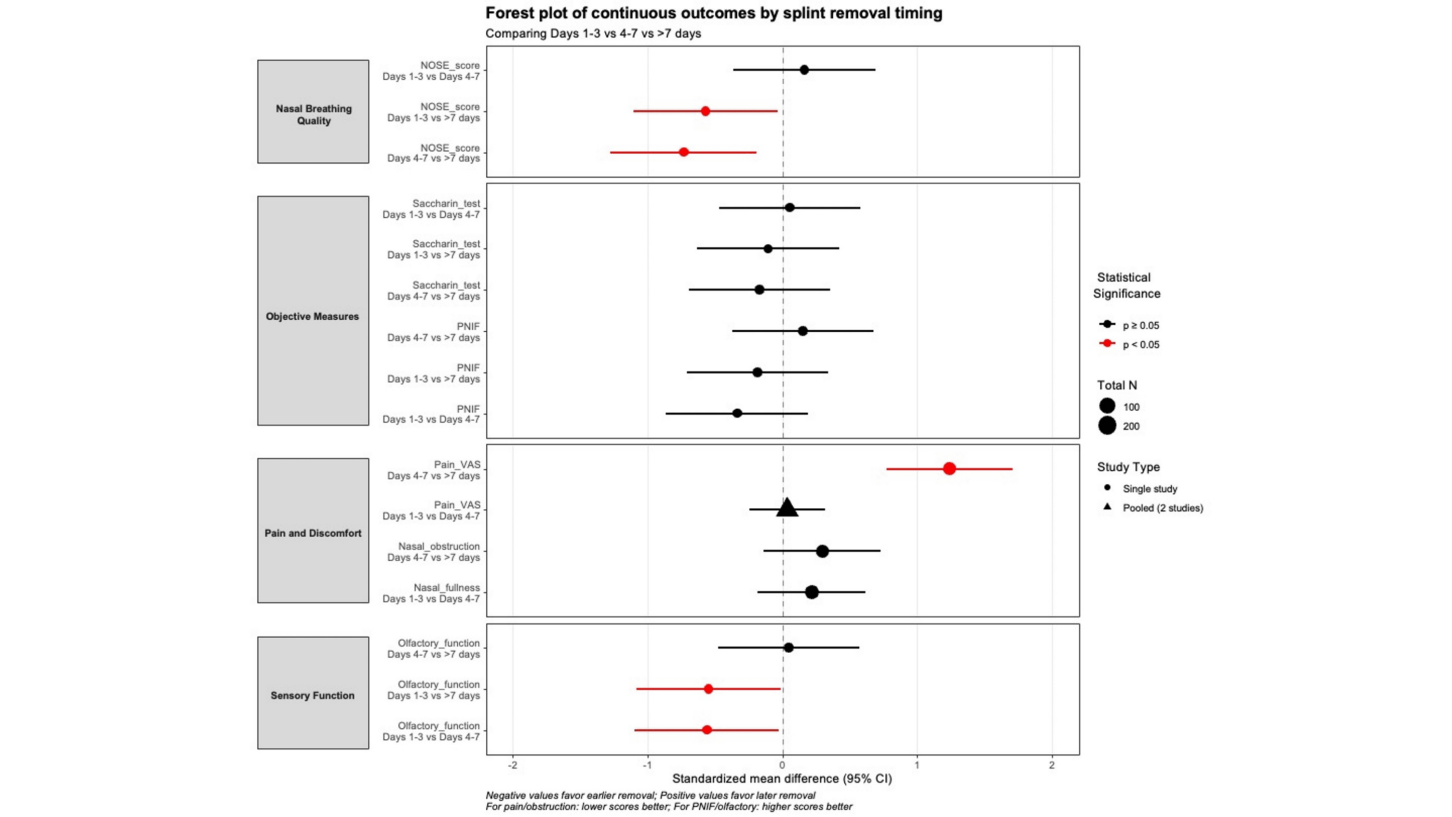
Single-study comparisons of continuous outcomes by splint removal timing This figure is original and was created by the authors from data extracted in this review; no previously published material has been reproduced.

The pooled analysis reveals several notable patterns across functional outcomes following septoplasty with different splint removal timings. A detailed summary of continuous postoperative outcomes by splint removal timing is presented in Table 3. Single‑study effect estimates for continuous outcomes across splint removal timings are shown in Figure 4. A substantial pain reduction was observed with late removal (>7 days) compared to intermediate timing (SMD 1.24, 95% CI 0.77‑1.71, p<0.001). The pooled analysis of two studies (n=209) found no difference between early (days 1‑3) and intermediate (days 4‑7) removal (SMD 0.04, 95% CI ‑0.24‑0.32, p=0.801, I^2^=0%), suggesting pain levels are comparable within the first week. Nasal fullness and obstruction showed similar, non‑significant trends (SMDs 0.22 and 0.30, respectively, both p>0.05).

**Table 3:** Meta-analysis of continuous outcomes by splint removal timing Note: Negative SMD values favor the first group in the comparison; positive values favor the second group. For pain/obstruction outcomes, lower scores are better. For PNIF and olfactory function, higher scores are better.SMD = (first group mean-second group mean)/pooled SD. Positive values indicate higher scores in the first group listed in each comparison; negative values indicate higher scores in the second group. For pain and NOSE outcomes, lower scores indicate better clinical status. For PNIF and olfactory function, higher scores indicate better clinical status. Heterogeneity statistics (Q, I^2^) are not applicable (-) for single-study comparisons. Bold p-values indicate statistical significance (P < 0.05). SMD: standardized mean difference; CI: confidence interval; Z: Z-statistic; Q: Cochran's Q statistic for heterogeneity; NOSE: Nasal Obstruction Symptom Evaluation; PNIF: peak nasal inspiratory flow; I^2^: percentage of variability due to heterogeneity

Outcome	Comparison	N Studies	Total N	SMD	95% CI	Z	P-value	Q (χ²)	Q P-value	I^2^
Pain VAS	Days 1-3 vs. Days 4-7	2	209	0.036	-0.244, 0.316	0.25	0.801	0.06	0.805	0.0%
	Days 4-7 vs. >7 days	1	95	1.238	0.771, 1.705	5.20	<0.001	-	-	-
Nasal fullness	Days 1-3 vs. Days 4-7	1	109	0.217	-0.183, 0.617	1.06	0.287	-	-	-
Nasal obstruction	Days 4-7 vs. >7 days	1	95	0.296	-0.138, 0.731	1.34	0.181	-	-	-
NOSE score	Days 1-3 vs. Days 4-7	1	56	0.163	-0.362, 0.688	0.61	0.543	-	-	-
	Days 1-3 vs. >7 days	1	56	-0.569	-1.104, -0.035	-2.09	0.037	-	-	-
	Days 4-7 vs. >7 days	1	56	-0.733	-1.274, -0.192	-2.66	0.008	-	-	-
PNIF	Days 1-3 vs. Days 4-7	1	56	-0.336	-0.863, 0.192	-1.25	0.212	-	-	-
	Days 1-3 vs. >7 days	1	56	-0.184	-0.709, 0.341	-0.69	0.493	-	-	-
	Days 4-7 vs. >7 days	1	56	0.150	-0.375, 0.675	0.56	0.575	-	-	-
Saccharin test	Days 1-3 vs. Days 4-7	1	56	0.054	-0.470, 0.578	0.20	0.839	-	-	-
	Days 1-3 vs. >7 days	1	56	-0.107	-0.631, 0.418	-0.40	0.690	-	-	-
	Days 4-7 vs. >7 days	1	56	-0.170	-0.695, 0.355	-0.64	0.525	-	-	-
Olfactory function	Days 1-3 vs. Days 4-7	1	56	-0.561	-1.095, -0.027	-2.06	0.039	-	-	-
	Days 1-3 vs. >7 days	1	56	-0.547	-1.080, -0.013	-2.01	0.045	-	-	-
	Days 4-7 vs. >7 days	1	56	0.049	-0.475, 0.573	0.18	0.854	-	-	-

Contrary to pain outcomes, NOSE scores were reported in a single study of 56 patients. Early removal showed significantly better NOSE scores compared to late removal (SMD -0.57, 95% CI -1.10 to -0.04, p=0.037). Intermediate removal also showed significantly better scores than late removal (SMD -0.73, 95% CI -1.25 to -0.20, p=0.008). Early vs. intermediate removal showed no significant difference (SMD 0.16, 95% CI -0.27 to 0.59, p=0.543).​

No significant differences emerged in objective assessments. PNIF measurements showed negligible effects across all comparisons (all SMDs <0.34, all p>0.20), while mucociliary clearance via saccharin transit time remained unchanged (all SMDs <0.18, all p>0.50). Early removal was associated with significantly lower Connecticut Chemosensory Clinical Research Center (CCCRC) odor scores compared to intermediate removal (SMD -0.56, p=0.039) and late removal (SMD -0.55, p=0.045), indicating greater impairment in smell. There was no significant difference between intermediate and late removal (SMD 0.05, p=0.854). These moderate effect sizes indicate that maintaining splints for at least four days may be necessary to preserve smell function during the initial healing period.

Categorical Outcomes

The pooled ORs for postoperative complications by splint removal timing are summarized in Table 4. The pooled analysis of postoperative complications revealed low event rates overall. There was insufficient evidence to detect a difference between splint removal timings, likely due to low event rates and imprecise estimates (wide CIs). For bleeding, early removal (days 1‑3) compared to intermediate removal (days 4‑7) showed higher but non‑significant odds (OR 1.89, 95% CI 0.58‑6.15, p=0.293, I^2^=0.0%). For infection, no statistically significant difference was observed between early and intermediate removal (OR 2.48, 95% CI 0.44-14.08, p=0.306).

**Table 4:** Meta-analysis of postoperative complications by splint removal timing Note: Only comparisons with ≥2 studies are shown. All pooled comparisons showed no statistically significant heterogeneity (I^2^=0.0%). Septal perforation showed higher odds with early removal (p=0.059), but this was not statistically significant. OR: odds ratio; CI: confidence interval; Z: Z-statistic; Q: Cochran's Q statistic for heterogeneity; I^2^: percentage of variability due to heterogeneity

Outcome	Comparison	N Studies	Total N	Events	OR	95% CI	Z	P-value	Q (χ^2^)	Q P-value	I^2^
Bleeding	Days 1-3 vs. Days 4-7	5	403	9	1.89	0.58-6.15	1.10	0.293	1.26	0.739	0.0%
Infection	Days 1-3 vs. Days 4-7	3	251	4	2.48	0.44-14.08	1.13	0.306	0.29	0.589	0.0%
Adhesions	Days 1-3 vs. Days 4-7	4	347	3	3.50	0.44-27.60	1.16	0.234	0.00	-	0.0%
Septal hematoma	Days 1-3 vs. Days 4-7	5	403	4	2.11	0.42-10.65	0.98	0.368	0.73	0.693	0.0%
Septal hematoma	Days 4-7 vs. >7 days	2	151	0	0.68	0.04-11.12	-0.27	0.788	0.07	0.789	0.0%
Septal perforation	Days 1-3 vs. Days 4-7	3	265	5	5.69	0.94-34.44	1.89	0.059	0.30	0.862	0.0%

In the case of adhesions, early removal showed higher odds than intermediate removal, but this difference was not statistically significant (OR 3.50, 95% CI 0.44-27.60, p=0.234). Results were imprecise, with wide CIs.

For septal hematoma, early versus intermediate removal showed no significant difference (OR 2.11, 95% CI 0.42‑10.65, p=0.368, I^2^=0.0%), and intermediate versus late removal (>7 days) also showed no effect (OR 0.68, 95% CI 0.04‑11.12, p=0.788, I^2^=0.0%).

Finally, septal perforation showed a numerically higher odds with early removal (OR 5.69, 95% CI 0.94-34.44, p=0.059), though this was not statistically significant and the estimate was imprecise due to low event rates and wide CIs.

Discussion

Evidence from a single study (n=95) suggests that late (>7 days) splint removal is associated with significantly higher pain scores compared to intermediate (days 4-7) splint removal (SMD + 1.24, 95% CI 0.77-1.71, p<0.001). While limited to one trial, this observation is consistent with the histopathological timeline of mucosal inflammation. Tan et al. demonstrated that inflammatory infiltration, edema, and epithelial disruption peak within three to five days post-splint placement, gradually resolving after one week. By delaying splint removal beyond this acute inflammatory window, patients experience reduced pain as mucosal stabilization progresses and tissue tensile strength increases [14]. This protective effect reflects the temporal dynamics of nasal mucosal wound healing. During the first 72 hours post-septoplasty, the mucosa undergoes acute inflammation with neutrophil infiltration, vascular permeability, and tissue edema. Splints physically separate opposing mucosal surfaces and reduce shear forces during this vulnerable period. By days 5-7, edema begins to resolve, epithelial migration accelerates, and early collagen deposition strengthens tissue integrity. Removing splints during the acute inflammatory phase (days 1-3) exposes immature, mechanically fragile mucosa to airflow turbulence and pain-inducing receptor stimulation, whereas removal after inflammatory resolution (≥7 days) occurs when nociceptive signaling has diminished. These findings align with Campbell et al., who reported fewer postoperative complaints with splints retained over five days, and Malki et al., who observed reduced discomfort when splints remained in place for 7-10 days. However, Arslan et al. found no significant pain score differences between day 3, day 5, and day 7 removal groups in 120 patients [15,16,12]. This discrepancy may reflect differences in pain assessment tools (visual analog scale (VAS) vs. numerical rating scale), timing of measurement (immediate vs. 24-hour post-removal), patient populations (age, baseline pain tolerance), or analgesic protocols. Although statistical heterogeneity was absent (I^2^=0), such methodological variability may not be fully captured by I^2^, underscoring the need for standardized pain assessment methodologies in septoplasty research. Beyond timing, splint material properties significantly influence postoperative pain - a potential confounder in interpreting timing-based comparisons. Wadhera et al. demonstrated that silastic intranasal splints produced less pain and fewer complications compared to traditional nasal packing (Merocel, vaseline gauze), attributed to the splints’ smooth surface, reduced mucosal contact pressure, and allowance of nasal breathing [17-20]. Similarly, Acıoğlu et al. compared Merocel tampons with vaseline gauze in 119 patients and found significantly higher pain scores (VAS: 6.2±1.4 vs. 4.1±1.2, p<0.001) and bleeding rates (18% vs. 7%, p<0.05) with Merocel, reflecting its greater expansion pressure against traumatized mucosa [21]. These findings highlight that postoperative pain is not only influenced by the duration of intranasal support but also by the materials that are used.

Early splint removal was associated with significantly higher subjective obstruction scores compared to intermediate and late removal, indicating a paradoxical finding that reflects competing physiological processes. While splints stabilize septal alignment, they also occupy intranasal volume and restrict nasal airflow in the immediate postoperative period when submucosal edema remains pronounced (peak at postoperative days 5-7). Early splint removal eliminates this mechanical obstruction, providing immediate subjective breathing relief despite incomplete mucosal healing. Patients perceive the absence of the splint as improvement regardless of residual septal deviation or mucosal inflammation. However, although early removal was associated with higher point estimates for certain complications, these differences did not reach statistical significance, and the corresponding CIs were wide, indicating substantial imprecision. A prospective comparison by Bayram et al. in 84 patients directly examined this timing question, finding no significant differences in VAS pain or PNIF scores between day 3, 5, and 7 removal (both p>0.05); however, NOSE scores were significantly worse in the earliest removal group (day 3) compared with both day 5 and day 7 (p=0.005 and p=0.026, respectively), with no difference between day 5 and day 7 (p=0.487) [9]. These findings, together with our pooled analyses, support the cautious conclusion that removal during the intermediate period may provide an optimal balance between minimizing complications and maintaining symptomatic relief, although the overall certainty of evidence remains low.

Long-term outcomes following septoplasty show sustained symptom improvement and patient satisfaction. A meta-analysis by Alessandri-Bonetti et al. (35 studies) reported a pooled baseline NOSE score of 68.1 with a mean reduction of -48.8 at six months (95% CI -54.6 to -42.9), reflecting a large, clinically meaningful improvement [22]. Similarly, Fearington et al. found durable benefit, with 75.4% of patients reporting satisfaction or improvement at a mean follow-up of 27 months [23]. However, several studies observed mild deterioration in subjective outcomes over time despite stable objective measures, suggesting that perceived benefit may diminish modestly. This highlights the potential role of optimal splint-timing strategies in enhancing early recovery and influencing long-term symptom durability.

Preoperative symptom severity significantly predicts the magnitude of postoperative obstruction improvement, though the pattern of improvement varies by measurement context. Hytönen et al. demonstrated in a prospective Finnish cohort (n=188) that disease-specific nasal symptoms improved markedly on Sino-Nasal Outcome Test (SNOT-22) (mean reduction 4.11 points, p=0.000045, n=126), with severe baseline symptoms yielding substantially greater gains compared with mild symptoms [24]. Notably, baseline severity predicted SNOT-22 improvement independently; however, this study revealed a paradoxical finding in generic quality of life measured by 15D, which worsened after septoplasty (baseline 0.949 to 0.928, p<0.001), particularly in older patients (age >55 years, p<0.01). This paradox suggests that disease-specific symptom improvement does not automatically translate to broader health-related quality of life gains, particularly in the aging population. In contrast, Stewart et al., in the sole study reporting NOSE scores (n=59), focused on satisfaction outcomes, finding that patients with severe preoperative obstruction (baseline NOSE 67.5) experienced significant improvement at three months (postoperative NOSE 23.1, p < 0.0001) sustained at six months, with very high patient satisfaction and decreased medication use [25]. Together, these converging findings demonstrate that severe preoperative obstruction predicts both the greatest disease-specific improvement and the highest postoperative satisfaction, while mild baseline symptoms yield smaller absolute gains and lower perceived benefit. Clinically, these observations from prior studies highlight the importance of baseline symptom severity in patient selection and preoperative counseling, particularly when setting expectations for individuals with mild obstruction.

The heterogeneity in postoperative pain, functional recovery, and complication rates observed across splint removal timing groups likely reflects unmeasured patient-specific and anatomical variables inadequately captured in the included trials. Mucosal thickness and hydration status represent critical baseline variables affecting splint-tissue interaction. Mucosal thickness varies substantially among individuals and determines the contact pressure exerted by a standardized splint thickness (typically 0.25-0.5 mm). Patients with baseline thin or dehydrated mucosa experience greater relative splint pressure against epithelial surfaces, potentially accelerating pain perception and inflammatory edema. Conversely, those with naturally thicker, better-hydrated mucosa tolerate the same splint with minimal pressure-related irritation. Early splint removal may provide disproportionate benefit for thin-mucosa patients by reducing pressure-induced pain and crusting, while intermediate-to-late removal optimizes healing stabilization in thick-mucosa patients, where pressure is less problematic. Environmental factors such as climate and humidity may further modulate mucosal hydration baselines, potentially explaining some geographic variation in optimal timing preferences. Nasal anatomy and baseline airway obstruction further modify timing effects. Patients with severe septal deviation, enlarged turbinates, or narrow nasal passages experience increased airflow turbulence and mechanical stress on the healing mucosa even with splint protection in situ. The splint itself occupies limited intranasal volume in already-obstructed airways, intensifying the sensation of nasal blockage during the acute postoperative period (days 1-7 when edema peaks). These anatomically disadvantaged patients report greater relief upon early splint removal despite increased complication risk, creating a patient-centered dilemma between safety and symptomatic comfort. Conversely, patients with relatively patent nasal anatomy experience less obstruction burden and may tolerate extended splint retention for healing optimization without excessive discomfort [7,15,16].

Early splint removal showed numerically higher odds of septal perforation compared with intermediate removal (OR 5.69, 95% CI 0.94-34.44; p=0.059; I^2^=0%), but this difference did not reach statistical significance, and the wide CI reflects imprecision due to low event rates. Premature splint removal may impair vascular resilience to airflow and nasal movement, resulting in local ischemia and subsequent septal perforation. Similar numerically higher perforation risks with earlier removal have been described by Arslan et al., Bayram et al., and Ozdogan et al., although these individual studies also did not demonstrate statistically significant differences [12-14]. Early removal was associated with higher odds of mucosal adhesion formation relative to intermediate removal, although this difference was not statistically significant (OR 3.50, 95% CI 0.44-27.60; p=0.234; I^2^=0%). A large tertiary referral center study of 5,639 septoplasty patients demonstrated overall low complication rates (3.42%), with adhesions, septal hematoma, and prolonged healing more frequent in combined septoplasty and turbinoplasty procedures involving extensive mucosal trauma, highlighting that baseline surgical extent is an important modifier independent of timing alone [3]. Mucosal crusting was more commonly observed during the intermediate removal period. This may reflect mucosal dryness and transient surface instability as healing transitions from the inflammatory phase to remodeling. Karataş et al. prospectively compared splint removal timing (days 5, 7, and 10) and found decreased mucosal crusting and synechia with longer splint retention, while postoperative pain and nasal obstruction diminished by postoperative day 3 regardless of timing [13]. These findings underscore that appropriate splint retention duration minimizes tissue injury and preserves mucosal integrity, particularly after extensive surgical manipulation. Although splint retention timing clearly influences mucosal healing, postoperative care factors also substantially affect crusting risk. Inadequate saline irrigation (<2 irrigations/day), poor patient adherence, and splint material (porous Merocel vs. smooth silastic) have been associated with increased crusting severity. Arslan et al. reported increased crusting in the late removal group (>7 days) despite reduced pain, highlighting that crusting is multifactorial and not determined by timing alone [12]. These observations underscore that minimizing crusting requires an integrated approach combining optimal timing, irrigation intensity, splint material selection, and adherence to postoperative care protocols.

These results carry important clinical implications. With respect to subjective nasal breathing assessed using the NOSE score, the available evidence is severely limited and of very low certainty. No statistically significant differences were observed in NOSE scores for: early versus late splint removal (based on a single study, n=56), or intermediate versus late splint removal (based on limited evidence, primarily from one or very few studies). Both comparisons were rated as very low certainty of evidence due to high risk of bias, very serious imprecision, inconsistency, indirectness (all studies conducted in Turkey), and restriction to a single-country dataset. Although early splint removal may theoretically reduce the duration of foreign-body sensation, our pooled estimates only suggest higher odds of mucosal adhesions and septal perforation while the tissues remain fragile and incompletely healed, and these differences did not reach statistical significance. Splint removal during the intermediate period appears to permit progressive mucosal healing while preserving adequate nasal airflow, and may offer a more favorable balance between symptomatic relief and potential complications; however, this interpretation is based on low‑certainty, hypothesis‑generating evidence and should be viewed with caution. Late splint removal is associated with reduced postoperative pain in limited data and may attenuate certain inflammatory responses; however, the prolonged presence of a foreign body and associated nasal obstruction may compromise overall patient satisfaction.

Certainty of Evidence Summary

We assessed the certainty of evidence for key outcomes using a structured approach considering risk of bias, imprecision, inconsistency, indirectness (all studies from Turkey), and publication bias. Certainty was rated as high, moderate, low, or very low. The detailed certainty of evidence ratings for each outcome are summarized in Table 5.

**Table 5:** Certainty of evidence summary The certainty of evidence was low for postoperative pain and very low for all other outcomes, primarily due to imprecision, single-study comparisons, and geographic restriction to Turkey. NOSE: Nasal Obstruction Symptom Evaluation; PNIF: Peak Nasal Inspiratory Flow; SMDs: standardized mean differences

Outcome	Comparisons	Certainty	Rationale
Postoperative pain	Early vs. late; early vs. intermediate; intermediate vs. late	Low	Moderate risk of bias in 4/6 studies; pooled SMDs imprecise; single-country evidence
NOSE scores	Early vs. late; intermediate vs. late	Very low	Single study (n=56); high risk of bias; very imprecise
Olfactory function	Early vs. intermediate	Very low	Single study; high risk of bias; very imprecise
PNIF	Early vs. late	Very low	Single study; unclear risk of bias; very imprecise

As a recommendation, a single standardized protocol for splint removal timing is unlikely to be universally effective. Instead, timing should be individualized based on each patient's clinical course, healing progression, and symptom burden. Subjective measures, such as patient feedback and reported complications in this review, were more sensitive to timing than objective assessments, such as PNIF or olfactory tests. This highlights the need for clinicians to use a holistic approach, considering not only quantitative outcomes but also patient-reported experience.

There are several strengths in this systematic review and meta‑analysis. It is, to current knowledge, the first quantitative synthesis that focuses on the effect of intranasal splint removal timing following septoplasty, addressing a clinically significant but previously unresolved question. The reliability of the results was reinforced by the inclusion of both randomized and observational comparative studies, along with subgroup analyses and random‑effects modeling. Moreover, the study assessed a comprehensive range of outcomes, both subjective and objective, providing a balanced assessment of postoperative recovery and patient comfort.

However, this systematic review has several limitations. First, most outcomes were evaluated only in the early postoperative period, so the long‑term impact of splint removal timing remains uncertain. Second, all included studies were conducted in Turkey, which may limit generalizability to other populations, surgical practices, and health‑care settings. Third, heterogeneity in splint materials, surgical techniques, follow‑up schedules, and outcome definitions likely contributed to variability in effect estimates. Finally, several included observational studies were at moderate to serious risk of bias, particularly with respect to confounding and selection, which reduces the overall certainty of the evidence.

All included studies originated from Turkey, raising questions about external validity. However, clinical practices in Turkey and Saudi Arabia share notable similarities that may support the applicability of these findings locally. Both countries utilize predominantly silicone splints, preferred over older plastic/acrylic materials due to flexibility and reduced mucosal pressure, with postoperative protocols often emphasizing saline nasal irrigation and short courses of prophylactic antibiotics. Patient demographics also overlap, with similar rates of septal deviation etiology (trauma, congenital) and climate-related factors influencing nasal mucosa healing. While subtle variations exist, such as potentially higher humidity in coastal Saudi regions (Jeddah) versus inland Turkey affecting crusting/bleeding rates, or differing follow-up intensity, the overall patterns of postoperative care and splint use appear comparable, which may enhance the contextual relevance of these results to similar Middle Eastern settings [26].

Future research should focus on high‑quality multicenter RCTs that directly compare clearly defined early, intermediate, and late removal intervals using standardized, validated outcome measures, including both patient‑reported and objective assessments. Studies incorporating longer follow‑up, detailed reporting of splint materials and postoperative care protocols, and exploration of patient‑level modifiers such as mucosal condition, comorbidities, and healing capacity are needed to guide truly individualized timing decisions.

## Conclusions

In conclusion, intranasal splint removal timing appears to influence several short-term postoperative outcomes after septoplasty, although the certainty of evidence is limited. While early removal (days 1-3) improves subjective nasal breathing, it risks increased complications, including septal perforation and adhesions. Intermediate removal (days 4-7) may offer a favorable balance, minimizing discomfort while allowing adequate mucosal stabilization. Late removal (>7 days) reduces pain but decreases patient satisfaction. Given patient variability, individualized removal timing based on healing progression and symptom severity is recommended rather than universal protocols. High-quality multi-center trials with standardized outcome measures and extended follow-up are needed to establish definitive guidelines.

## References

[1] Van Egmond MM, Rovers MM, Hendriks CT, van Heerbeek N (2015). Effectiveness of septoplasty versus non-surgical management for nasal obstruction due to a deviated nasal septum in adults: study protocol for a randomized controlled trial. Trials.

[2] Watters C, Brar S, Yapa S (2022). Septoplasty. StatPearls [Internet].

[3] Dąbrowska-Bień J, Skarżyński PH, Gwizdalska I, Łazęcka K, Skarżyński H (2018). Complications in septoplasty based on a large group of 5639 patients. Eur Arch Otorhinolaryngol.

[4] Genç E, Ergin NT, Bilezikçi B (2004). Comparison of suture and nasal packing in rabbit noses. Laryngoscope.

[5] Kotler R, Wahl K, Lee KJ (2020). Solving the problem of post-operative airway obstruction in nasal/sinus surgery. Arch Otolaryngol Rhinol.

[6] Ivanova PP, Iliev G (2023). Nasal packing in septal surgery: a narrative review. Cureus.

[7] Aksoy E, Serin GM, Polat S, Kaytaz A (2011). Removing intranasal splints after septal surgery. J Craniofac Surg.

[8] Jung YG, Hong JW, Eun YG, Kim MG (2011). Objective usefulness of thin silastic septal splints after septal surgery. Am J Rhinol Allergy.

[9] Dag I, Acar M, Sakallioglu O, Catli T, San T, Cingi C (2014). Influence of surface properties of Merocel® (polyvinyl acetal) and silicone nasal splints on biofilm formation. Eur Arch Otorhinolaryngol.

[10] Karatas A, Pehlivanoglu F, Salviz M, Kuvat N, Cebi IT, Dikmen B, Sengoz G (2016). The effects of the time of intranasal splinting on bacterial colonization, postoperative complications, and patient discomfort after septoplasty operations. Braz J Otorhinolaryngol.

[11] Ozdogan F, Ozel HE, Esen E, Yuce T, Eyisarac S, Genc S, Selcuk A (2016). Optimal time for intranasal splint removal after septoplasty: a prospective clinical study. Eur Arch Otorhinolaryngol.

[12] Bayram Ö, Hacı C, Akpınar YA, Paksoy M, Coşkun SC, Açıkalın RM (2022). Assessment of ideal duration of intranasal splint use after septoplasty: a prospective randomized clinical study. KBB - Forum.

[13] Arslan S, Yıldırım H, Çobanoğlu B, Işık AÜ, Bahadır O (2024). Impact of intranasal splint removal time on postoperative complications after septoplasty. Niger J Clin Pract.

[14] Tan M, Kalcioglu MT, Sahin N, Bayindir T, Samdanci E, Filiz A (2015). Assessment of mucosal changes associated with nasal splint in a rabbit model. Braz J Otorhinolaryngol.

[15] Campbell JB, Watson MG, Shenoi PM (1987). The role of intranasal splints in the prevention of post-operative nasal adhesions. J Laryngol Otol.

[16] Malki D, Quine SM, Pfleiderer AG (1999). Nasal splints, revisited. J Laryngol Otol.

[17] Wadhera R, Zafar N, Gulati SP, Kalra V, Ghai A (2014). Comparative study of intranasal septal splints and nasal packs in patients undergoing nasal septal surgery. Ear Nose Throat J.

[18] Ardehali MM, Bastaninejad S (2009). Use of nasal packs and intranasal septal splints following septoplasty. Int J Oral Maxillofac Surg.

[19] Bresnihan M, Mehigan B, Curran A (2007). An evaluation of Merocel and Series 5000 nasal packs in patients following nasal surgery: a prospective randomised trial. Clin Otolaryngol.

[20] Bingöl F, Budak A, Şimşek E, Kılıç K, Bingöl BÖ (2017). Comparison of early-period results of nasal splint and Merocel nasal packs in septoplasty. Turk Arch Otorhinolaryngol.

[21] Acıoğlu E, Edizer DT, Yiğit Ö, Onur F, Alkan Z (2012). Nasal septal packing: which one?. Eur Arch Otorhinolaryngol.

[22] Alessandri-Bonetti M, Costantino A, Cottone G (2023). Efficacy of septoplasty in patients with nasal obstruction: a systematic review and meta-analysis. Laryngoscope.

[23] Fearington FW, Awadallah AS, Hamilton GS 3rd, Olson MD, Dey JK (2024). Long-term outcomes of Septoplasty with or without turbinoplasty: a systematic review. Laryngoscope.

[24] Hytönen ML, Lilja M, Mäkitie AA, Sintonen H, Roine RP (2012). Does septoplasty enhance the quality of life in patients?. Eur Arch Otorhinolaryngol.

[25] Stewart MG, Smith TL, Weaver EM, Witsell DL, Yueh B, Hannley MT, Johnson JT (2004). Outcomes after nasal septoplasty: results from the Nasal Obstruction Septoplasty Effectiveness (NOSE) study. Otolaryngol Head Neck Surg.

[26] Hassan ME, Hashem HF, Abdelsamei AA, Mohamdy AA (2021). Intranasal splint removal after septal surgery: optimum timing. Pan Afr Med J.

